# Haloperidol-Induced Laryngeal Dystonia: A Case Report on an Antipsychotic-Associated Airway Emergency

**DOI:** 10.7759/cureus.74761

**Published:** 2024-11-29

**Authors:** Lorenzo E Guani, Angelica Arshoun, Andrew S Murdock, Eduardo D Espiridion

**Affiliations:** 1 Psychiatry, Drexel University College of Medicine, West Reading, USA; 2 Psychiatry, Reading Hospital - Tower Health, West Reading, USA

**Keywords:** antipsychotic medication, critical airway, emergency, haloperidol-induced, laryngeal dystonia

## Abstract

Acute dystonia is a neurological condition characterized by involuntary muscle contractions that can affect various parts of the body. It is commonly triggered by the use of antipsychotic medications, especially within the first few days after administration. Respiratory acute laryngeal dystonia, a particularly severe form of this condition and a very subtype of laryngeal dystonia, can lead to respiratory distress and airway obstruction if not promptly treated. This case report describes a 23-year-old male who developed acute laryngeal dystonia within 24 hours of receiving haloperidol for agitation. The patient presented with hoarseness, difficulty swallowing, and progressive respiratory distress, eventually requiring emergent intubation due to airway compromise. This case underscores the need for healthcare providers to recognize and promptly manage rare but potentially life-threatening side effects of antipsychotic medications. Particular attention must be directed toward patients with risk factors for developing extrapyramidal reactions. Early intervention is crucial to prevent progression to airway obstruction and respiratory failure.

## Introduction

Acute dystonia is characterized by involuntary muscle contractions affecting the extremities, face, neck, abdomen, pelvis, or larynx, which can occur in sustained or intermittent patterns. Dystonia can be classified by the body part it affects, the age of onset, or other characteristics. Laryngeal dystonia may be respiratory, but the majority is an adductor spasmodic dystonia that affects the muscles used for speech but may not affect respiration and does not lead to airway obstruction. These contractions can lead to painful and uncomfortable postures for patients [1]. Acute dystonia is an extrapyramidal symptom (EPS) often triggered by antipsychotic medications, typically manifesting within 48 hours to five days after administration [1,2]. Approximately 3%-10% of individuals receiving antipsychotics experience an acute dystonic reaction, with a higher incidence observed in those prescribed higher-potency agents, such as haloperidol [2]. Respiratory acute laryngeal dystonia, a specific type of acute dystonia, is a spasmodic contraction of the larynx that can potentially be life-threatening if left untreated [3]. It often presents with symptoms of dysphonia, dysphagia, dyspnea, and stridor [2]. Current research has not shown a conclusive mechanism of action for this reaction; however, it is postulated that the imbalance in the basal ganglia between acetylcholine, an inhibitor of movement, and dopamine, a stimulator of movement, could be responsible for these reactions [1]. Treatment of dystonic reactions aims to adjust the dopaminergic-cholinergic imbalance with the use of first-line agents, diphenhydramine and benztropine [1]. Our case report illustrates a patient who developed laryngeal dystonia within 24 hours of administration of haloperidol and who subsequently required emergent intubation due to respiratory distress. The aim of this case study is to document an adverse effect of haloperidol and other antipsychotics and provide insight into this rare, yet severe, reaction.

## Case presentation

This is a 23-year-old male with a past medical history of hypertension, gastroesophageal reflux, and cannabis hyperemesis syndrome taking ondansetron as needed for nausea. The day prior to his admission, the patient presented to the emergency department with complaints of vomiting after ingestion of psychedelic mushrooms and marijuana. For safety reasons, the patient was sedated with haloperidol 10mg, diphenhydramine 50 mg, and lorazepam 2 mg because he was agitated, screaming, and fighting with staff. He was successfully calmed and re-oriented. The emergency medicine provider wanted to keep him for observation due to the administration of haloperidol and the potential adverse effects of the psychedelic mushrooms. Despite conversations with the physician, he left against medical advice. The following day, he returned to the emergency department with complaints of vomiting, locking of the mouth and jaw, and an inability to swallow or drink. He reported severe pain and a feeling of swelling in his mouth. There was no stridor noted, but the patient had weak vocalization and hoarseness.

On initial physical examination, the patient was overweight with a BMI of 29.16 kg/m^2^, afebrile, tachypneic with respiratory rates greater than 20 per minute, hypertensive at 147/118, tachycardic with heart rates over 90 beats per minute, and had poor dentition with dental tenderness and gingival swelling. His mental status examination showed inattention with labile and inappropriate affect in addition to being uncooperative and withdrawn. His blood work was significant for a potassium of 3.2 mmol/L and white blood cell count of 22,300 cells/µL, creatine kinase of 1,033 IU/L; however, other laboratory values were within normal limits (Tables 1, 2).

**Table 1:** Bloodwork upon initial presentation to the emergency department

Lab	Value	Reference range
Sodium	140 mmol/L	136-145 mmol/L
Potassium	3.2 mmol/L	3.5-5.1 mmol/L
Chloride	106 mmol/L	100-110 mmol/L
Bicarbonate	22 mmol/L	20-31 mmol/L
Anion gap	12 mmol/L	2-10 mmol/L
BUN	10 mg/dL	9-23 mg/dL
Creatinine	0.78 mg/dL	0.73-1.18 mg/dL
Glucose	101 mg/dL	74-99 mg/dL
Calcium	10.2 mg/dL	8.7-10.4 mg/dL
Alkaline phosphatase	70 IU/L	46-116 IU/L
Albumin	5 g/dL	3.4-5 g/dL
Total protein	8.1 g/dL	5.7-8.2 g/dL
Creatine kinase	1,033 IU/L	46-171 IU/L
White blood cells	22.3 x 10^3^/µL	4.8-10.8 x 10^3^/µL
Eosinophil count	0.01 x 10^3^/µL	0.04-0.54 x 10^3^/µL
Hemoglobin	14.0 g/dL	14-17.5 g/dL
Platelets	295 x 10^3^/µL	130-400 x 10^3^/µL

**Table 2:** Arterial blood gas results

Lab	Value	Reference range
pH	7.389	7.350-7.450
PCO2	35.6 mmHg	35-48 mmHg
PO2	108.0 mmHg	83.0-108.0 mmHg
HCO3	21.5 mmol/L	21-28 mmol/L
Base excess	-2.9 mmol/L	-2.0 - 3.0 mmol/L
O2 sat	+99.3%	94.0%-98.0%

Additionally, urinalysis showed a specific gravity of +1.045, 160 mg/dL of ketones, large amounts of blood with over 100 red blood cells per high-power field, and 100 mg/dL of protein; as well, urine toxicology was positive for THC but negative for other substances (Tables 3, 4). 

**Table 3:** Urine testing upon initial presentation to the emergency department POC - point of care

Lab	Value	Reference range
Color	Dark yellow	Yellow, dark yellow, light yellow, colorless
Appearance	Clear	Clear, cloudy, other
pH	6.0	5.0-8.0
Specific gravity	+1.045	1.005-1.028
Glucose	Negative	Negative
Ketones	+160 mg/dL	Negative
Bilirubin	Negative	Negative
Blood	Large	Negative
Protein	100 mg/dL	Negative
Urobilinogen	1.0 mg/dL	0.2-1.0 mg/dL
Leukocyte esterase	Trace	Negative
Nitrite	Negative	Negative
Hyaline casts	0-2/LPF	0-2/LPF
Blood, POC	+100/HPF	0-2/HPF
Epithelial cells	0-2/HPF	0-2/HPF
White blood cells	0-5/HPF	0-5/HPF
Bacteria	None	None

**Table 4:** Toxicology screening results PCP - phenylcyclohexyl piperidine, THC - tetrahydrocannabinol

Lab	Value	Reference range
Opiates	Negative	Negative
Amphetamines	Negative	Negative
Methadone	Negative	Negative
Cocaine	Negative	Negative
Barbiturates	Negative	Negative
Benzodiazepine	Positive	Negative
PCP	Negative	Negative
THC	Positive	Negative
Oxycodone	Negative	Negative
Fentanyl	Negative	Negative

**Figure 1:**
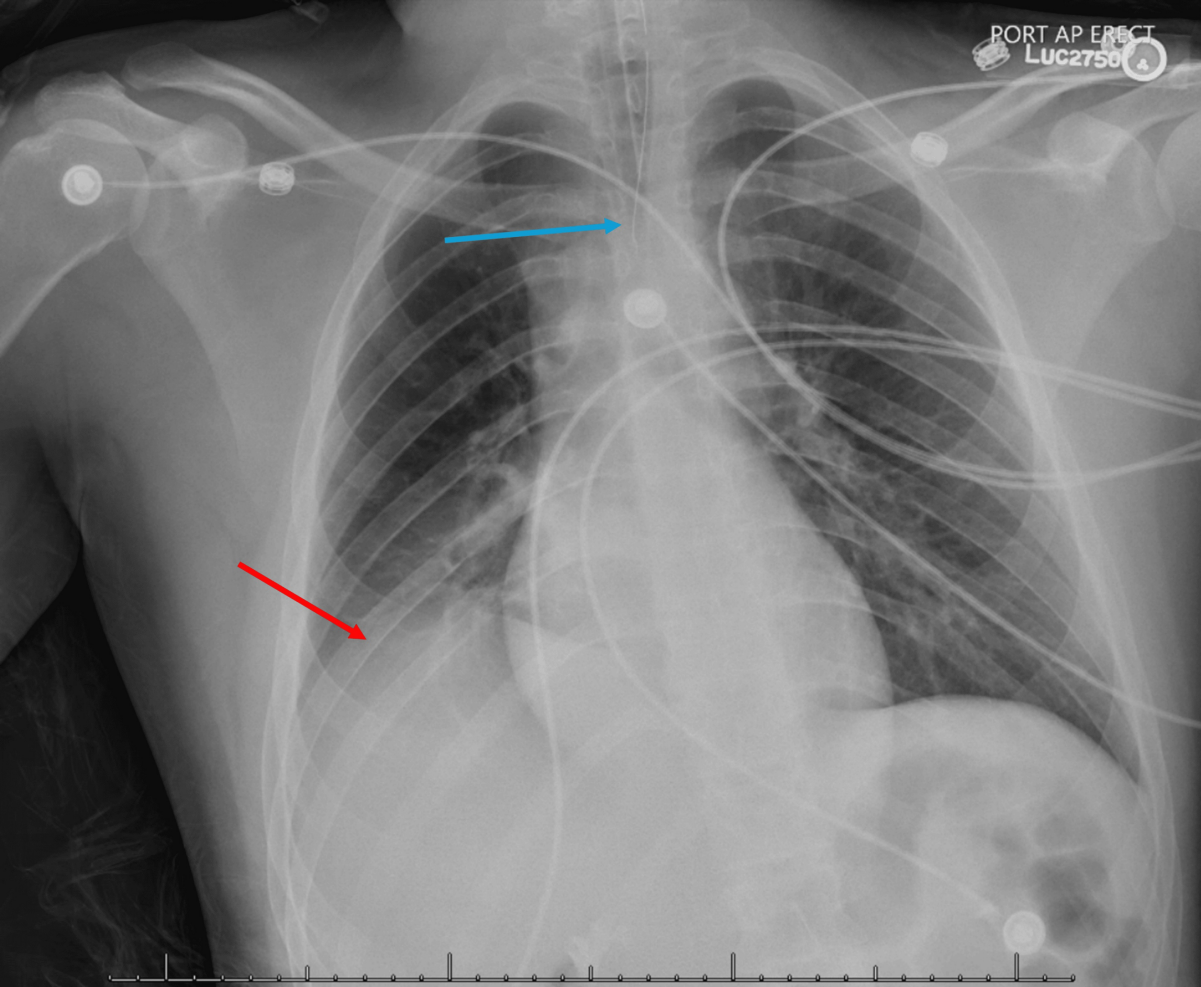
Frontal view of the chest demonstrates a normal cardio-mediastinal silhouette without focal consolidation, pleural effusion, or pneumothorax. There is a stable right hemidiaphragm elevation (red arrow). Endotracheal tube terminates at the superior margin of the clavicular heads, 4.6 cm above the carina (blue arrow).

Shortly after his current presentation to the ED, he began drooling secretions and developed respiratory decompensation after expressing a tightening sensation in his throat. Due to the rapid progression of his difficulty breathing, he ultimately required emergent intubation due to worsening facial, oral, and laryngeal muscle swelling and spasms. His intubation was complicated but was successfully done using a 6.0 endotracheal tube after a 7.0 could not be passed. Upon examination of his larynx, severe laryngeal extrinsic and intrinsic muscle swelling and spasms were noted. The vocal cords tightened and close together. Chest x-rays taken at the time showed proper placement of the tube without upper airway obstruction (Figure 1).

Additionally, he was given diphenhydramine, epinephrine, and dexamethasone to help reduce his laryngeal extrinsic and intrinsic muscle swelling and dystonic spasms. He was stabilized and maintained on ventilation for five days. During this time, he was sedated with propofol and continued to be treated with dexamethasone and diphenhydramine.

A collateral history collected from his significant other disclosed that he previously received haloperidol for the management of agitation and violence secondary to substance use. At that time, he had a similar, although less severe, reaction. However, there was no significant history of any other psychiatric treatment or diagnosis in the past.

After excluding other etiologies, such as anaphylaxis, angioedema, and retropharyngeal abscess, this patient was diagnosed with laryngeal dystonia due to haloperidol use. He was extubated and stabilized on day five of his admission without further complications.

## Discussion

Haloperidol, a first-generation antipsychotic, is widely used for its effectiveness in treating a variety of psychiatric conditions and acute agitation, such as in this patient. However, its role as a potent D2 receptor antagonist brings about a risk of EPS, including but not limited to dystonic reactions [1]. Dystonia is defined as a movement disorder characterized by involuntary muscle contractions [1]. Varying forms of dystonia exist, with some being classified on the basis of the number of regions that are impacted within the body. The mechanism underlying haloperidol and other antipsychotic-induced dystonia is likely related to dopamine receptor blockade within the nigrostriatal pathway of the basal ganglia, leading to disruption in muscle tone and movement coordination [3]. The disruption of this pathway can specifically lead to disinhibited contraction of laryngeal muscles, causing spasmodic closure and airway compromise [3]. Although the exact mechanism is not fully understood, one study using positron emission tomography found that animal models treated with haloperidol had a higher incidence of dystonic reactions and a higher occupancy of D2 receptors, suggesting that blockage of these receptors is closely related to the development of dystonia [4]. Specifically, the authors of the study found that an occupancy of D2 receptors of 78% or more was a likely threshold for dystonia [4]. Even a low dose of haloperidol, such as less than five grams per day, was found to reach the aforementioned threshold of D2 blockage [4].

The incidence of isolated dystonia in the general population is documented to be 35.1 in 100,000 leading to focal muscle spasm or contraction in specific regions of the body [5]. Acute laryngeal dystonia is particularly rare and potentially life-threatening, as it can lead to airway obstruction requiring emergent intubation. The general presentation of laryngeal dystonia varies from subtle findings, such as mild throat discomfort, to fulminant airway obstruction [2]. This patient’s initial presentation with hoarseness of the voice following administration of haloperidol may have been an early indicator that subsequent dystonic issues were impending.

Numerous cases of haloperidol-induced dystonia have been previously documented, but most involve less severe dystonic reactions, such as extraocular dystonia, torticollis, and limb dystonia [6-9]. However, a recent systematic review of 45 cases of laryngeal dystonia underscores the prevalence of laryngeal dystonia following antipsychotic administration and suggests the need for heightened awareness among practicing clinicians [3]. Such reactions tend to be more prevalent in younger patients, male patients, and patients who have a history of dystonic reactions to other offending agents [10-12]. In this case, it is possible that this patient had a dystonic reaction during a previous administration of haloperidol, additionally, his sex and age put him at higher risk for developing a dystonic reaction to offending agents.

In terms of differential diagnoses, a potential etiology of this patient’s symptoms includes medication-related anaphylaxis due to the rapid onset of respiratory distress, laryngeal swelling, and a history of similar symptoms occurring following a previous administration of haloperidol. Despite throat closure being a prominent feature of anaphylactic shock, drug-related anaphylactic reactions typically occur within minutes, as opposed to hours such as in this patient [13]. Additionally, anaphylactic reactions are generally associated with urticaria, and symptoms of shock including hypotension with a systolic blood pressure being less than 90 mmHg, tachycardia, altered mental status, anuria, and stridor, among others [13]. The patient was specifically noted to not have stridor, and his vital signs at the time of intubation were significant for only tachypnea and tachycardia. Other types of allergic reactions, such as laryngeal angioedema, are more likely differentials for this patient's symptoms. The presentation of laryngeal edema can mimic laryngeal dystonia in that both conditions can present with airway compromise [14]. Additionally, both conditions may be incited by haloperidol administration. The patient’s past reaction to haloperidol could further support either diagnosis. Ultimately, an allergic response to haloperidol would likely be associated with other key features of an allergic reaction: eosinophilia, urticaria, pruritus, and a history of asthma, eczema, or other conditions that increase the suspicion of an allergic reaction [14]. He did not have eosinophilia or a past medical history of atopy or presentation suggestive of an allergic response outside his larynx (Table 1). While these findings do not entirely exclude laryngeal angioedema as a diagnosis, they paint a picture that makes laryngeal dystonia appear to be the more likely etiology.

Other differential diagnoses could include retropharyngeal abscess. The presentation would be similar to this patient with symptoms including difficulty swallowing and respiratory distress [15]. Abscess formation can be incited by different etiologies including upper respiratory tract infections, trauma to the posterior pharynx, or dental pathology [15]. Therefore, it is possible this patient’s poor dentition led to abscess formation. Further, abscesses cause an inflammatory process that could explain this patient’s leukocytosis, but we would also expect the patient to have a fever and neck stiffness [15]. On x-ray imaging, we would see a hypodense lesion in the retropharyngeal space [16]. Retropharyngeal abscesses are best assessed with lateral views of the neck; however, these were not taken for this patient.

Treatment for laryngeal dystonia involves correcting the dopaminergic-cholinergic imbalance through several medications, as well as discontinuing the use of the offending antipsychotic [1]. Immediate treatment includes diphenhydramine or benztropine. These drugs are used due to their anticholinergic properties and central nervous system penetration. In emergent cases, such as this one, intravenous administration of diphenhydramine is preferred over other routes due to its faster onset of effects [1]. Benztropine, on the other hand, has an equal onset of effects regardless of intravenous or intramuscular administration [1]. Another treatment includes the use of benzodiazepines. Intravenous benzodiazepine administration is indicated if patients do not experience complete or only partial improvement in their symptoms [1]. Additionally, if a patient appears to be in respiratory distress or fails to properly oxygenate, intubation may be considered to protect the patient from further respiratory decline as was required in this patient [1]. Botulinum toxin has been used in respiratory acute laryngeal dystonia. This has been used in patients with abductor involvement as well as abductor involvement [17].

In patients with cannabis use disorder, cannabinoid withdrawal could contribute to the dystonic reaction by altering neurological dopamine signaling [18]. Studies have shown that cannabis withdrawal leads to decreased secretion of dopamine in the mesolimbic system [18]. It is plausible that cannabinoid withdrawal might also alter dopaminergic signaling in the basal ganglia as endocannabinoids have been shown to directly and indirectly influence dopamine secretion in this region [19]. Therefore, cannabis withdrawal might increase a patient's susceptibility to developing dystonic reactions. Regarding polypharmacy, in the setting of cannabis-induced hyperemesis, it is important to avoid the combination of first-generation antipsychotics coupled with dopaminergic antiemetics that might increase a patient’s risk for developing dystonic reactions. For patients using substances recreationally, it is also important to assess where they access or purchase their drugs. While this history was not assessed for this case, cannabis obtained from non-legal sources has the potential to be laced with other drugs leading to unexpected adverse effects [20]. While the patient's substance use might not have directly contributed to his presentation, at the very least, it caused him to receive the haloperidol which led to his life-threatening respiratory failure.

## Conclusions

This case emphasizes the need for clinicians to be vigilant about potential EPS, particularly in patients with risk factors for dystonic reactions including younger age, male gender, and a prior history of extrapyramidal-related side effects to other medications. Given the critical nature of laryngeal dystonia, clinicians should be prepared to recognize and manage symptoms of upper airway obstruction in patients on haloperidol. Early signs may include stridor, dyspnea, and voice changes, which, if promptly identified, can allow for appropriate management with diphenhydramine or benztropine to prevent progression to full airway obstruction.
